# TLS data for cracks detection in building walls

**DOI:** 10.1016/j.dib.2022.108247

**Published:** 2022-05-05

**Authors:** Paulina Stałowska, Czesław Suchocki

**Affiliations:** Faculty of Civil Engineering, Environmental and Geodetic Sciences, Koszalin University of Technology, Śniadeckich 2, Koszalin 75-453, Poland

**Keywords:** Terrestrial laser scanner, TLS, Diagnostic measurements, Crack detection, Intensity

## Abstract

Nowadays, non-destructive remote measuring techniques are popular methods applied in civil engineering, and one of them is terrestrial laser scanning (TLS). The poor condition of buildings is a very significant aspect of the construction field. Symptoms such as cracks and other discontinuities of the building's wall can be detected using TLS [3], [4], [5], [1]. For the authors, it is important to exploit the full potential of this methodology. In addition to 3D spatial coordinates, terrestrial laser scanners also register radiometric information of laser beams reflected from scanned surfaces, so-called *intensity*. In the authors' opinion analyses of both types of information lead to more reliable cracks detection. The authors examined how the technical parameters of the scanner and the ‘geometric’ conditions of the measurement affect the results. The obtained results enabled the authors to explore possibilities and limitations of TLS and allowed them to develop a measurement methodology for detecting building wall cracks using a terrestrial laser scanner. The collected datasets contains point clouds from the measurements of special sample with cracks and measurements of real buildings in poor technical condition.

## Specifications Table

**Table:** 

Subject	Civil and Structural Engineering
Specific subject area	Remote Sensing, Non-Destructive-testing (NDT),
Type of data	LiDAR point cloud (files in the *.pts format were stored in the *.bin files supported by the CloudCompare open source software, https://www.danielgm.net/cc/), Image
How the data were acquired	The data were acquired using a phase-shift scanner Z + F IMAGER 5016.
Data format	Raw
Description of data collection	The tested samples with cracks were scanned from different distances and different incidence angle in laboratory and field condition.In the laboratory stage the specimen was tested from four distances (5 m, 10 m, 15 m and 20 m), and five angles of incidence (0 gon (0°), 20 gon (approx. 18°), 40 gon (approx. 36°), 60 gon (approx. 54°), and 80 gon (approx. 72°)) for each of these distances. In the field stage, three walls of the different buildings were tested.
Data source location	Institution: Koszalin University of TechnologyCity/Town/Region: KoszalinCountry: PolandLatitude and longitude (GPS coordinates) for collected samples/data:latitude: 54.200383, longitude: 16.19721building 1: latitude: 54.225188, longitude: 16.294588;building 2: latitude: *54.225298,* longitude: *16.292056;*building 3: latitude: *54.229438,* longitude: *16.296750;*
Data accessibility	Repository name: Mendeley dataData identification number: DOI: 10.17632/6f3trnj2ym.1Direct URL to data: https://data.mendeley.com/datasets/6f3trnj2ym/1
Related research article	P. Stałowska, C. Suchocki, M. Rutkowska, Crack detection in building walls based on geometric and radiometric point cloud information, Automtion Constr. DOI: 10.1016/j.autcon.2021.104065

## Value of the Data

• The data can be used by researchers to analyse possible application of TLS technology for crack detection in building and structures.
• The point clouds obtained under various measurement conditions provide the opportunity to plan an appropriate measurement strategy for a particular crack.
• The datasets provide radiometric and geometric information of the point clouds. That can be useful for the assessment which type of data is more useful for the particular crack case.
• The data can be exploited for analysis of the absorption and dispersion of the laser beam reflected from inhomogeneous surface wall, especially from cracks.

## 1 Data Description

The obtained data are point clouds (*.pts format), with the following structure:

X, Y, Z, RGB, Intensity (0 – 1)

Where:

X, Y, Z are 3D coordinates of points, unit [m]

RGB are information about the colour captured by digital camera.

Intensity is a relation between emitted and received signal power by TLS (radiometric information of point cloud).The datasets in question consist of two families of data. Datasets were obtained in the laboratory and in the field conditions by Z+F IMAGER 5016 scanner. A special specimen was used for laboratory tests, and real buildings with poor technical conditions were used for field tests. The names of the folders reflect the measurements conditions (the lab stage) or the object of research (the field stage). The structure of the name of the files is as follows:

XXgonYYm.bin - where XX stands for the angle of incidence (0, 20, 40, 60 and 80gon), and YY stands for the distance in meters from TLS to target (5, 10, 15 and 20m).

## 2 Experimental Design, Materials and Methods

As mentioned above, the research consist of two stages, in the laboratory and field conditions. For the laboratory stage, researchers prepared a specimen of white silicate with ten grooves from 1 mm to 10 mm widths, that simulated cracks. The field stage were performed on three buildings with cracks of different widths and depths. In both cases, the data were collected in similarly way using a phase-shift (PS) scanner Z + F 5016 IMAGER (manufactured in 2019). For that scanner, the diameter of the beam is 3.5 mm at the exit, and the beam divergence is 0.3 mrad. Other technical parameters of used scanner are shown in Table 1. All measurements were performed using the maximum scanning resolution (extremely high: 80000 pixel/360°).

**Table 1:** Technical characteristics of used scanner.

	Z + F IMAGER 5016
Type of Rangefinder	phase-shift
Wavelength	1500 nm
Scan Rate Points/sec	1,100,000
Range	365 m
Ranging Error	±1 mm + 10 ppm/m
Accuracy of Vertical and Horizontal Angle	14.4’’
Maximum Vertical / Horizontal Angular Resolution	0.00026° / 0.00018°

The operation of terrestrial laser scanner is based on the use of laser light to obtain information about geometric and radiometric parameters. The terrestrial laser scanner emits a laser beam by recording the horizontal (α) and vertical (φ) angles, and then the distance (s) is determined due to the reflection of the laser beam from the observed object. The acquired information is used to calculate the XYZ spatial coordinates of points in the local system of the scanner. In addition, terrestrial laser scanners record radiometric information of the returning laser beam so called *intensity*, which is defined as the ratio of the received energy power to the emitted energy power.

**Fig. 1:**
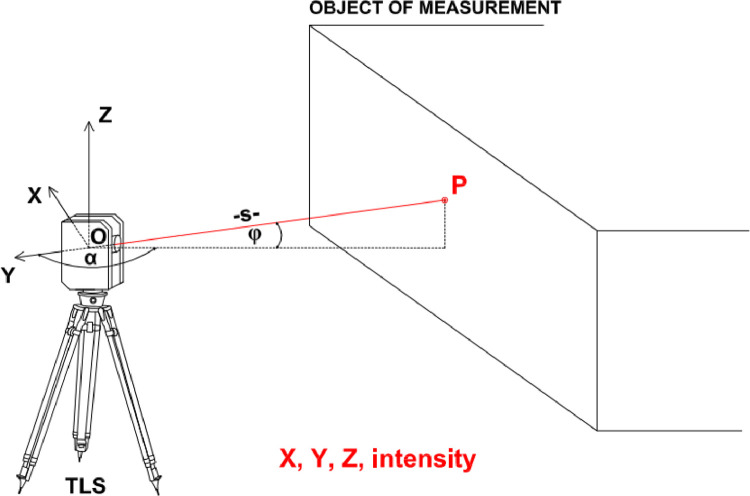
The scheme of the data collected by TLS.

A scheme of the data collected by TLS is shown in Fig. 1.

**Fig. 2:**
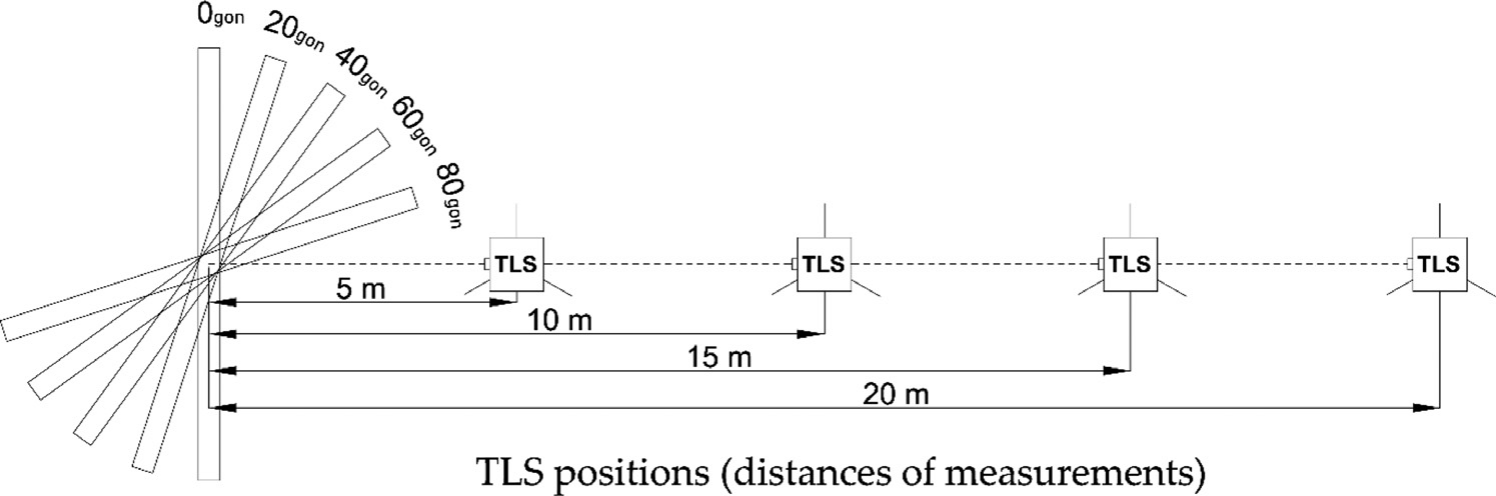
Schematic of the measurement methodology in laboratory conditions [1].

The lab stage was the basis for the research. The specimen was scanned from four distances (5 m, 10 m, 15 m and 20 m), and five angles of incidence (0 gon (0°), 20 gon (approx. 18°), 40 gon (approx. 36°), 60 gon (approx. 54°), and 80 gon (approx. 72°)) for each of these distances. A schematic of the measurement methodology under laboratory conditions is shown in Fig. 2.

The field stage constituted as a confirmation of some obtained results. Based on the walls of three different buildings in poor condition, some measurements conditions were verified. Object 1 was scanned under one measurement condition, with an angle of incidence and a distance of 7 gon (approx. 6.3°) and 13.8 m. Object 2 was scanned under two measurement conditions, with an angle of incidence and a distance of 2 gon (approx. 1.8°) and 5.1 m, and 44 gon (approx. 39.6°) and 8.9 m. The last, Object 3 was scanned only under one condition, with an angle of incidence of 26 gon (approx. 23.4°) and a distance of 3.5 m due to the surroundings of the object. The neighbouring buildings and the road in close vicinity made it impossible to scan Object 3 under other measurement conditions. It is worth to pointing out that such a situation is quite often encountered.

The measurements resulted in 20 point clouds in the laboratory, and 4 point clouds in the field conditions. For the post-processing of datasets and mapping of results the open-source CloudCompare software was used. All point clouds obtained in the laboratory conditions were registered to the same coordinate reference system based on special artificial Z + F profit targets.

**Fig. 3:**
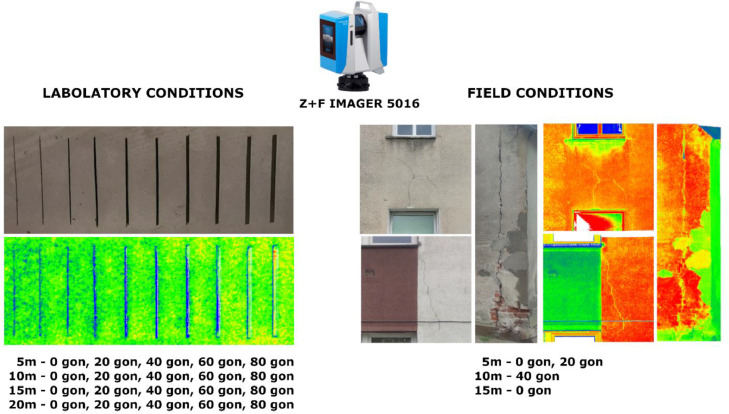
Overall concept of the study modified from Stałowska et al. [1].

General concept of the research and objects of the research are shown in Fig. 3.

## Ethics Statements

This work did not involve human subjects, animal experiments and data collected from social media platforms.

## CRediT Author Statement

**Paulina Stałowska:** Conceptualization, Methodology, Validation, Investigation, Resources, Writing – original draft, Writing – review & editing, Supervision, Visualization, Project administration; **Czesław Suchocki:** Conceptualization, Methodology, Software, Investigation, Resources, Data Curation, Writing – original draft, Writing – review & editing.

## Declaration of Competing Interest

The authors declare that they have no known competing financial interests or personal relationships that could have appeared to influence the work reported in this paper.

## Data Availability

TLS data for cracks detection in building walls (Original data) (Mendeley Data).
